# Investigation of Deep-Learning-Driven Identification of Multiple Sclerosis Patients Based on Susceptibility-Weighted Images Using Relevance Analysis

**DOI:** 10.3389/fnins.2020.609468

**Published:** 2020-12-18

**Authors:** Alina Lopatina, Stefan Ropele, Renat Sibgatulin, Jürgen R. Reichenbach, Daniel Güllmar

**Affiliations:** ^1^Medical Physics Group, Institute for Diagnostic and Interventional Radiology, University Hospital Jena, Jena, Germany; ^2^Michael-Stifel-Center for Data-Driven and Simulation Science Jena, Jena, Germany; ^3^Department of Neurology, Medical University of Graz, Graz, Austria; ^4^Center of Medical Optics and Photonics Jena, Jena, Germany

**Keywords:** convolutional neural network, deep learning, explainability, magnetic resonance imaging, multiple sclerosis, susceptibility-weighted imaging, interpretable AI, machine learning

## Abstract

The diagnosis of multiple sclerosis (MS) is usually based on clinical symptoms and signs of damage to the central nervous system, which is assessed using magnetic resonance imaging. The correct interpretation of these data requires excellent clinical expertise and experience. Deep neural networks aim to assist clinicians in identifying MS using imaging data. However, before such networks can be integrated into clinical workflow, it is crucial to understand their classification strategy. In this study, we propose to use a convolutional neural network to identify MS patients in combination with attribution algorithms to investigate the classification decisions. The network was trained using images acquired with susceptibility-weighted imaging (SWI), which is known to be sensitive to the presence of paramagnetic iron components and is routinely applied in imaging protocols for MS patients. Different attribution algorithms were used to the trained network resulting in heatmaps visualizing the contribution of each input voxel to the classification decision. Based on the quantitative image perturbation method, we selected DeepLIFT heatmaps for further investigation. Single-subject analysis revealed veins and adjacent voxels as signs for MS, while the population-based study revealed relevant brain areas common to most subjects in a class. This pattern was found to be stable across different echo times and also for a multi-echo trained network. Intensity analysis of the relevant voxels revealed a group difference, which was found to be primarily based on the T1w magnitude images, which are part of the SWI calculation. This difference was not observed in the phase mask data.

## Introduction

Multiple sclerosis (MS) is the most common neuroimmunological disease and causes a high demand on healthcare resources (Stenager, 2019). In addition to the necessary cost-intensive medication and ongoing care, expert knowledge and experience are required to diagnose the disease correctly. The McDonald criteria (Thompson et al., 2018) used to diagnose MS include the presence of clinical symptoms together with radiological signs. Although the disease pattern can be identified by magnetic resonance imaging (MRI) contrasts, there is a risk of clinical misinterpretation. The development of algorithms to automate the diagnosis of MS based on MRI data would make a valuable contribution in this regard.

Today, machine learning algorithms and in particular deep neural networks are making remarkable progress in biomedical image analysis, especially in supporting clinicians in decision making (Arbabshirani et al., 2018; Katzman et al., 2018). Regarding MS, most of these applications perform automated lesion segmentation based on both FLAIR and T_2_-weighted MR images or a combination of both (Brosch et al., 2016; Valverde et al., 2017; Aslani et al., 2019; Gabr et al., 2019). However, the presence of lesions is not always associated with the disease, and the lesion patterns can be quite heterogeneous and are not necessarily unique for MS (Filippi et al., 2019). Therefore, a more relevant issue now being addressed with deep neural networks is the classification of MS patients and healthy subjects directly from the data without prior lesion segmentation.

Recently, a few studies have focused on MS classification based on convolutional neural networks (CNNs) without lesion segmentation (Wang et al., 2018; Zhang et al., 2018; Marzullo et al., 2019). Zhang et al. (2018) have proposed a 10-layer CNN-PreLU-Dropout approach for identifying MS patients based on 2D T_2_-weighted axial MRI data that outperforms other modern MS identification approaches (Murray et al., 2010; Wang et al., 2016; Wu and Lopez, 2017; Ghirbi et al., 2018). Wang et al. (2018) have proposed an improved structure of the CNN-PreLU-Dropout approach (Zhang et al., 2018) by incorporating batch normalization, and stochastic pooling applied to the same data and achieved superior performance compared to the original method (Zhang et al., 2018). Marzullo et al. (2019) used the graph CNN model to classify MS patients into four clinical profiles (clinically isolated syndrome, relapsing-remitting, secondary-progressive, and primary-progressive) and to distinguish them from healthy controls. In contrast to the studies mentioned above, the latter was applied to structural connectivity information extracted from diffusion MRI data.

Although they have shown promising results, none of these approaches bring new insights into the radiological signs relevant to the diagnosis of MS. For medical diagnostics, understanding the decision-making process of a neural network is essential, not least to reduce the risks of clinical misinterpretation and to ensure appropriate treatment. Today, there are various possibilities in computer science to make neural networks more explainable. A group of these interpretability methods is used to generate attribution maps (heatmaps) that highlight features of the input image that affect the output (Simonyan et al., 2013; Zeiler and Fergus, 2014; Bach et al., 2015; Springenberg et al., 2015; Lapuschkin et al., 2016; Shrikumar et al., 2016, 2017; Kindermans et al., 2017; Smilkov et al., 2017; Sundararajan et al., 2017).

To the best of our knowledge, only one study has so far taken any steps to uncover CNN decisions in MS classification. Eitel et al. (2019) applied layer-wise relevance propagation (LRP) (Bach et al., 2015; Lapuschkin et al., 2016) to reveal image features captured with a proposed naive 3D CNN network for MS identification. Their analysis showed individual lesions, the location of the lesions, and some non-lesion areas as relevant input data compartments. However, conventional T_2_-weighted images, as used by Eitel et al. (2019), are usually only valuable in terms of lesion information, while other MR contrasts, such as susceptibility-weighted imaging (SWI) (Haacke et al., 2004), may show additional radiological signs relevant to MS. Newly established patterns in susceptibility-weighted images include the central vein sign (Lamot et al., 2017; Sparacia et al., 2018), iron depositions (Dal-Bianco et al., 2017; Yan et al., 2018), cerebral microbleeds (Zivadinov et al., 2016), and venous anatomy (Dal-Bianco et al., 2015; Öztoprak et al., 2016) and have the potential to indicate the presence of MS or to characterize the course of the disease. Signal intensity on SWI varies depending on tissue composition. Iron, for example, appears on SWI as a hypointense signal, whereas white matter demyelination appears hyper- or isointense (Chawla et al., 2018).

The aim of this study was, therefore, to identify MS patients using a CNN model based on SWI data and to investigate the classification strategy of the model using different attribution algorithms [LRP (Bach et al., 2015; Lapuschkin et al., 2016), DeepLIFT (Shrikumar et al., 2016, 2017), saliency maps (Simonyan et al., 2013)] and individual heatmaps indicating the contribution of a given voxel to the classification decision. The quality of the heatmaps was evaluated by means of perturbation analysis (Samek et al., 2017), as this technique does not require visual evaluation, which implicitly requires prior knowledge and diagnostic experience.

## Materials and Methods

### Data Acquisition and Preprocessing

Three-dimensional T_1_-weighted multi-echo gradient-echo images were acquired on a 3T MRI scanner (Prisma Fit, Siemens Healthineers, Erlangen, Germany) using a 20-channel head coil. The sequence parameters were as follows: α = 35°; TE_1–5_ = (8.12; 13.19; 19.26; 24.33; 29.40 ms); TR = 37 ms, matrix-size = 168 × 224; FOV = 168 mm × 224 mm; slice thickness = 1 mm; number of slices = 192. The entire database includes data from 184 patients with MS and 66 healthy subjects. 66 patient datasets were randomly selected to balance the number of patients and controls. All investigations were conducted in accordance with the Declaration of Helsinki on Ethical Principles for Medical Research Involving Human Subjects. The demographical characteristics of the two groups can be found in Table 1.

**TABLE 1:** Demographic information of MS and HC subjects included in the study.

	MS	HC	*t*-Test result (*p*-value)
Number of subjects	66	66	
Age in years (mean ± standard deviation)	39.94 ± 11.71	36.05 ± 11.72	0.06
Male/female	38/62%	47/53%	0.29

*The last column displays the *p*-value for the *t*-test for each attribute.*

Each MR dataset was preprocessed using the typical SWI routine (Haacke et al., 2004) for each echo time separately. This SWI preprocessing was implemented in Python using the following steps. First, *k*-space data, which were retrieved from magnitude and phase data, were filtered with a symmetric Hamming window (Blackman and Tukey, 1958) of size 128 × 128. By using complex division, the filtered reconstructed phase images were subtracted from the original phase images (homodyne filtering). To generate the phase masks (PMs) in the phase range −π to +π, positive phase values were set to one and negative values were scaled between zero and one. The PMs were multiplied four times with the corresponding magnitude images. Magnitude images were not corrected for intensity inhomogeneities prior to SWI computation to avoid spurious residuals of this procedure in the SWI images. We also assumed that the intensity inhomogeneity pattern should be similar between subjects and thus not relevant to the classification procedure. Finally, minimum intensity projections were computed in a sliding window manner over 14 consecutive slices. Thus, for each subject, five different 3D SWI (using the MRI data for each of the echoes separately) were reconstructed. For the single-echo experiments, we used SWI data reconstructed for a single echo, i.e., in section “Attribution Methods and Perturbation Analysis” we used SWI reconstructed for the TE_5_, and in section “ROI-Based Analysis” – for the TE_3_. For the multi-echo experiments in section “Population-Based Attributions,” five separate SWI data from each of the five TEs were used.

For each SWI scan volume, one single 2D projection in transverse orientation with its center at a predefined slice position and predefined echo time was selected as one sample for the resulting input dataset to the CNN. Moreover, we applied skull stripping to each 2D image (projection) and standardized the masked images to zero mean and unit variance. Finally, the dataset was split into training and test sets each containing 33 samples per class.

### CNN Model Architecture and Training

**FIGURE 1:**
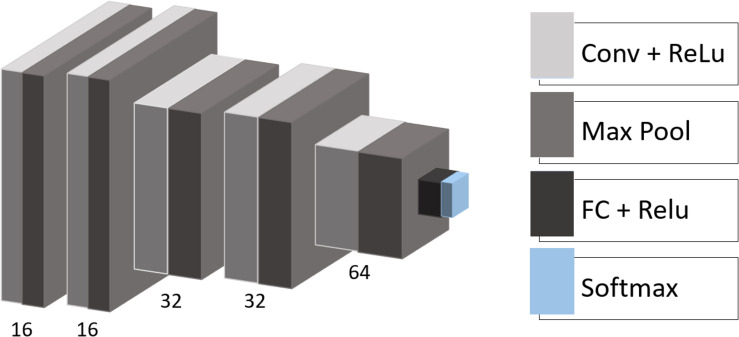
CNN architecture. Conv, convolutional layer; Max Pool, max-pooling operation; ReLU, rectified linear unit; FC, fully connected layer; Softmax, softmax layer.

We used the following architecture for the CNN with empirically adjusted hyperparameters (Figure 1): the model consists of five convolutional layers with rectified linear unit (ReLU) activation functions followed by max-pooling layers with a pooling window size of 2 × 2. The number of filters in the convolutional layers is equal to 16, 16, 32, 32, and 64 with a kernel size of 3 × 3. One fully connected layer (eight neurons) with ReLU activation and one output layer (two neurons) with soft-max activation complete the structure of the model. Besides, we applied dropout regularization to the output of the first and the last two max-pooling layers.

With each iteration during the training epoch, a batch of randomly augmented samples replaced the corresponding input batch of samples of the training set. This in-place augmentation technique was used to avoid overfitting and to increase robustness to the new data. We applied several data augmentation settings to our data, including image rotation between 20° and +20° around the center of the slice, horizontal and vertical shifting in the range between (−20; 20) and (−12; 12) voxels, respectively, scaling with factors between 0.7 and 1.0 as well as horizontal flipping. The data generator randomly transformed an image according to the predefined settings. The training was early stopped if there were no performance improvements to the model on the validation set, and the model with the best validation accuracy was saved and used for further analysis.

### Attribution Methods and Perturbation Analysis

To explain the decisions of our CNN model we rely on attribution methods that can be divided into local and global ones (Ancona et al., 2018). While local attributions illustrate how small changes to the input feature contribute to the output, global attributions represent the importance of a feature weighted relative to other input features. We used two publicly available global attribution methods, LRP (Bach et al., 2015; Lapuschkin et al., 2016) and DeepLIFT (Shrikumar et al., 2016, 2017), and compared them to saliency maps (Simonyan et al., 2013) as a local attribution method. All of these algorithms operate layer-wise in a backward fashion. The LRP algorithm (Bach et al., 2015; Lapuschkin et al., 2016) decomposes the output classification score into the relevance of the corresponding input voxels. Similarly to LRP, DeepLIFT (Shrikumar et al., 2016, 2017) assigns relevance to the input values, which explains the difference in the output with respect to reference input values. Saliency maps (Simonyan et al., 2013) are computed by propagating the partial derivative of the target output with respect to the input features.

The attribution maps (heatmaps) were generated for all subjects in the test set. The produced maps were analyzed with perturbation analysis (Samek et al., 2017), a promising method that does not require human judgment and ground truth. The attribution algorithms as well as the perturbation analysis were implemented using iNNvestigate (Alber et al., 2019). In perturbation analysis, information from the image is perturbed region by region from most to least relevant according to the attribution map. The target output score of the classifier is affected by this perturbation and quickly drops if highly relevant information is removed. The faster the classification score drops, the better an attribution method is capable to identify the input features responsible for correct classification. To numerically assess changes in the classification score over the perturbation steps, for each method we compute the area over the perturbation curve (AOPC):

$$\text{AOPC}=\frac{1}{L+1}\,\left\langle \sum_{k=0}^{L}f\,\left(x^{\left(0\right)}\right)-f\,\left(x^{\left(k\right)}\right)\right\rangle ,$$

where *L* is the number of perturbation steps; *x*^(0)^ is the non-perturbed input image and *f* (*x*^(0)^) is the output classification score for this input; *x*^(^*^*k*^*^)^ is the input image after *k* perturbation steps and *f* (*x*^(^*^*k*^*^)^) is the corresponding classification score. ⟨^∗^⟩ denotes averaging over all images in the test data set.

### Population-Based Attributions

Based on the perturbation analysis, we chose one attribution method for detailed investigation. To identify brain regions relevant for the classification across MS and HC populations in the test set, we first registered all subjects to the selected reference subject using SimpleElastix (Marstal et al., 2016). All transformations were computed on the SWI images and then applied to the heatmaps. Next, each heatmap was smoothed with a Gaussian kernel of size 5 × 5 voxels. Finally, we averaged heatmaps over correctly predicted MS and HC.

In addition, we tested the stability of the chosen method by computing average heatmaps for a model trained on adjacent slice positions and on the same slice position, but with different echo time. To check the consistency of the relevance patterns, we ran multi-echo experiments by modifying the network’s architecture such that it takes distinct echoes through multiple channels. Similarly, to the single-echo case, we produced average heatmaps to evaluate characteristic spatial distributed relevance patterns.

### ROI-Based Analysis

**FIGURE 2:**
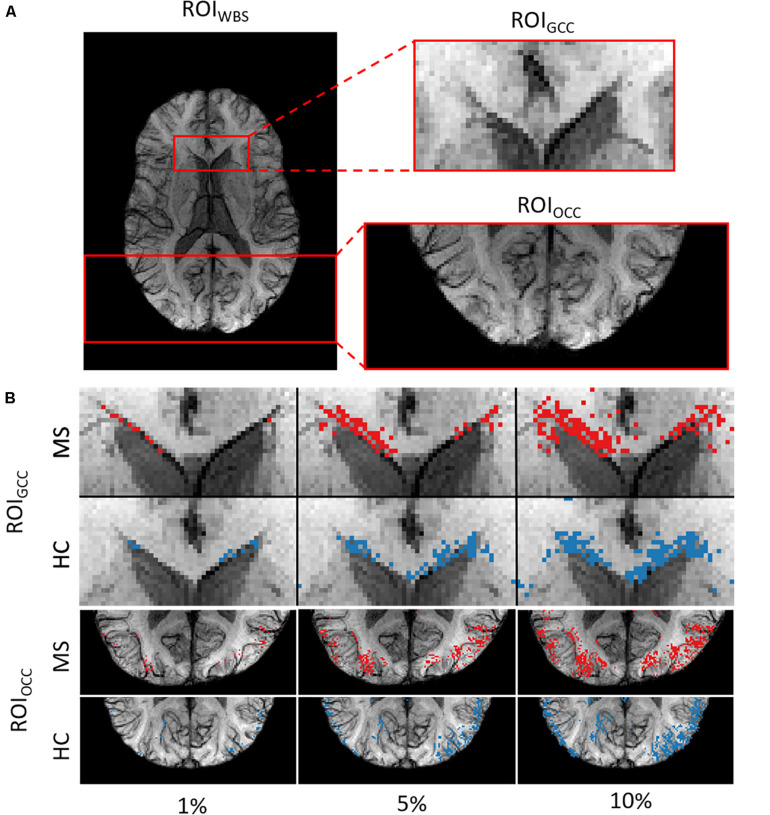
**(A)** Example of the three ROIs for a single subject. **(B)** ROI_*GCC*_ (upper row) and ROI_*OCC*_ (lower row) for the same subject in a close-up view with different amounts of included voxels for analysis.

We picked out three regions-of-interest (ROI) (Figure 2A) to analyze the potential distinguishability between MS and HC based merely on the relevant voxels in these regions. In our hypothesis, important areas may contain voxel information sufficient to distinguish patients from healthy subjects. We suppose that a straightforward statistical analysis of this information can lead to new findings on MS markers.

We used two ROIs with the relevance pattern consistent over different TEs in the average heatmaps and the whole-brain area. Moreover, for each ROI we analyzed different percentage coverage of relevant voxels (1, 5, 10, 50, and 100%). As an input sample, we used the mean value of the SWI voxels, which correspond to the highest positive relevant heatmap values for the MS class and the lowest negative relevance values for the HC class in a specific ROI with the respective percentage coverage setting. The same kind of evaluation was performed for the T1w magnitude data and the computed PMs, which were used for the SWI computation, separately.

To analyze the significance of the differences between two groups of subjects (MS and HC), for each configuration of the contrast, ROI and relevance percentage, we computed *p*-values using a two-sample *t*-test and Glass’ Δ effect sizes.

## Results

### Comparison of Attribution Algorithms

We used the perturbation analysis to compare heatmaps computed with the LRP, the DeepLIFT, and the saliency map algorithms. For the LRP, the ε-rule with a numerical stabilizer ε = 1, and for the DeepLIFT, reveal-cancel rule were selected as backpropagation rules. We choose these propagation rules based on the heatmap quality criterion (less noisy). The AOPC values were calculated over the 66 images from the test data set. In each perturbation step, ten regions of size 10 × 10 voxels were replaced by random values from the uniform distribution. Perturbation order is defined by heatmap values, starting from the most positive relevant values for prediction to the most negative ones. We replaced the first 130 regions in 13 steps resulting in 34.5% of the image being perturbed. We assume that this sufficiently perturbs the brain area, which contains information important for the classification.

**FIGURE 3:**
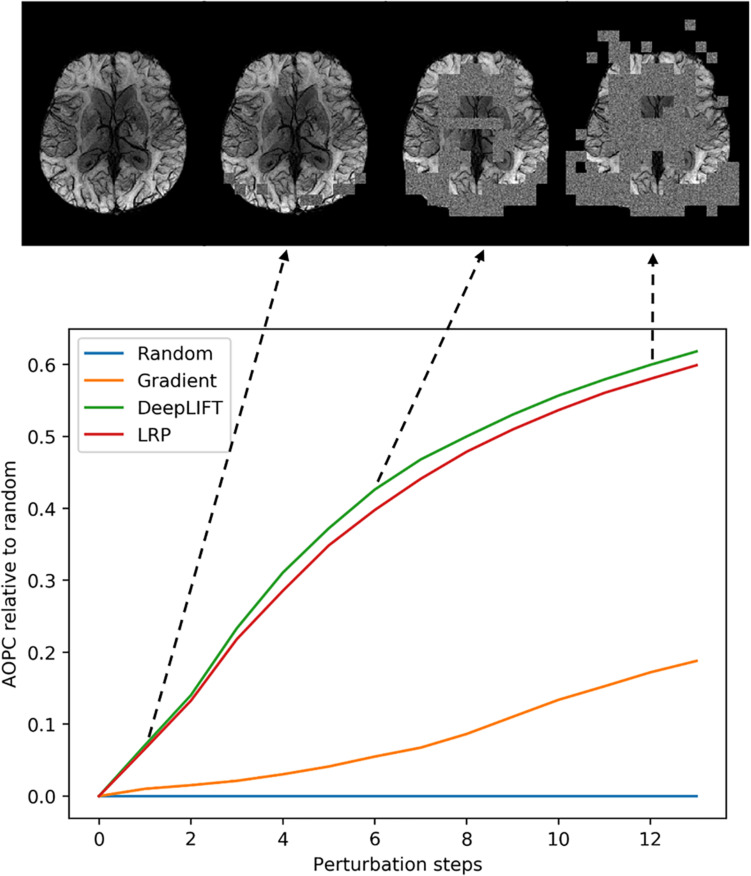
Averaged (66 subjects) AOPC plotted over perturbation steps (0–13) for four different relevance assessment methods (including random order, which is used as reference). In each perturbation step, 10 regions of size 10 × 10 were substituted with reference values. The upper part of the figure shows four different stages in the perturbation analysis for a single MS subject.

In Figure 3, AOPC curves are shown for each method. It can be seen that both LRP and DeepLIFT have the most significant AOPC values with DeepLIFT performing slightly better after a few perturbation steps. Since the saliency map aims to identify local relevance only, it performs much worse but still outperforms the random baseline. Based on the AOPC curves, we consider DeepLIFT as the preferable method and used it for further qualitative analysis.

### Individual Heatmaps

**FIGURE 4:**
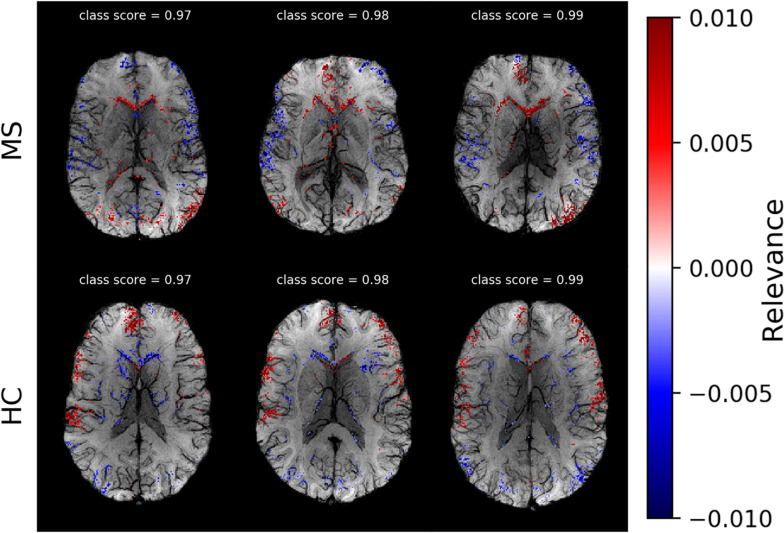
Voxel-wise relevance analysis results plotted as heatmaps on top of original SWI contrast images for three correctly classified MS and three HC subjects, respectively. Red shows the relevance of voxel positions that speak for the correct class and blue against it. The class probability for the correctly assigned class is stated at the top of each individual data set.

After selecting the algorithm, we analyzed DeepLIFT heatmaps for subjects in the test data set who had the highest classification score. In Figure 4, we show heatmaps, which are overlaid on the corresponding SWI images for three correctly classified MS patients, and three correctly classified HC. The heatmaps were threshold for the first and the last percentile of the relevance values and display only the highest positive and the lowest negative relevant voxels. The positive (red) and negative (blue) values indicate the relevance of a particular input voxel for or against a predicted class, respectively. In both groups of subjects, the attribution is mainly detected at the location of veins and voxels adjacent to these veins.

### Average Heatmaps

**FIGURE 5:**
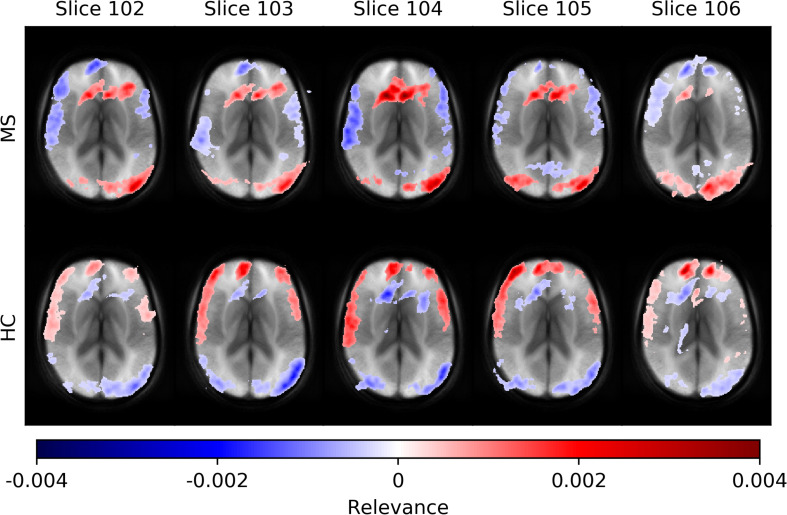
Averaged voxel-wise relevance heatmaps for correctly classified subjects over a range of consecutive axial slice positions. The upper row shows the averages for subjects from the MS group and the lower for the HC group.

In Figure 5, we show average heatmaps for all correctly classified MS patients and HC in the test set using CNN models trained on different slice positions. The heatmaps are thresholded, retaining only 5% of the highest positive and 5% of the lowest negative relevance voxels. We overlaid them with the average of all test subjects. Relevant features were mainly found in the brain periphery (cortical band) and showed a stable trend across different slice positions. Areas of voxels with positive relevance for one class are predominantly negative for the opposite class. For MS patients, positive relevance was observed in the lower parts of the brain and around central veins, while negative relevance was seen in the anterior brain parts.

The comparison of networks trained with data computed from different echo times and for the multi-echo case showed only minor deviations in model performance (accuracy) and average classification score. Neither a specific echo time nor the multi-echo model showed a distinctly deviating high or low performance. The corresponding numerical values are listed in Table 2.

**TABLE 2:** List of observed model performance (accuracy) and average classification scores for the analysis using networks trained and evaluated for different echo times as well as for the multi-echo case.

Single-echo					Multi-echo

Echo 1	Echo 2	Echo 3	Echo 4	Echo 5	
**Model performance (accuracy)**					
0.95	0.91	0.92	0.92	0.94	0.92
**Average classification score (MS/HC)**					
0.91/0.88	0.93/0.87	0.91/0.92	0.95/0.94	0.94/0.94	0.91/0.89

**FIGURE 6:**
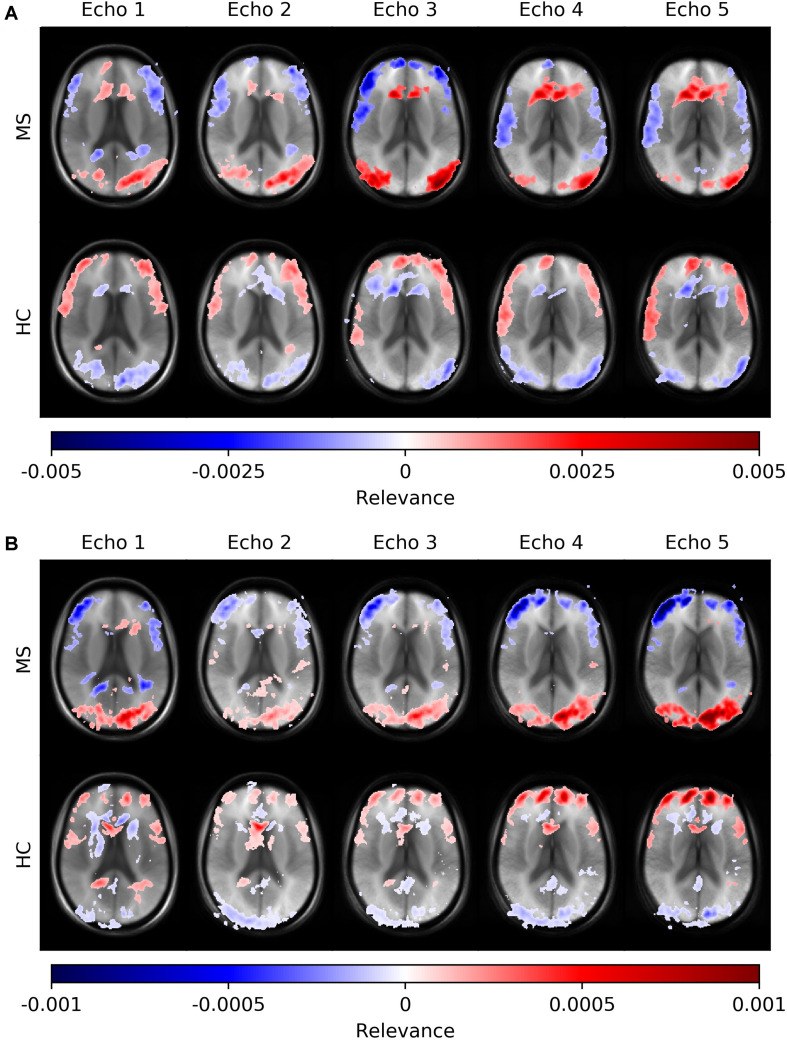
Averaged voxel-wise relevance heatmaps for correctly classified subjects from the test set across different echo times. The network was trained on single-echo data **(A)** and multi-echo data **(B)**.

Figure 6 depicts average heatmaps across different echo times for a fixed slice position for models trained on single-echo data (A) and multi-echo data (B). In the single-echo case, each average heatmap was generated with a trained network on data of the corresponding echo time; the most relevant areas were found in the frontal region around the anterior horn of the ventricles and in the occipital region, where for the latter region the right hemisphere was more pronounced. The highest positive relevance was found in the occipital region for echo #3 and in the anterior region for echo #5. Areas with the most negative relevance were observed in a cortical band in medial and anterior locations. The characteristics along this band changed with different echo times. The average relevance heatmaps for the HC group also showed different patterns for the different echo times. Areas with positive relevance for the MS group showed negative relevance for the HC group (anterior horn of the ventricles and the occipital region) and in a reversed relation for the anterior medial cortical band. The characteristics of the pattern for the HC group also changed over different echo times. In the multi-echo case, each average heatmap was generated on a trained network on data for all different echo times and the relevance patterns showed differences in comparison to the single echo case as well as between different echo times. The relevance of the anterior horn of the ventricles was significantly reduced, while the occipital region was similarly pronounced for the MS group. The maximal relevance was observed for echo #5 in the MS group. For the HC group in the multi-echo trained network, the relevance pattern was less characteristic in comparison to the MS group and the single-echo case.

### ROI-Based Numerical Analysis

In the last experiment, we performed an ROI-based numerical analysis based on the information provided by the relevance analysis for three different rectangular ROIs (Figure 2A): genu of the corpus callosum (GCC), occipital cortex (OCC), whole-brain slice (WBS), and for three separate image contrasts (SWI, T1w magnitude, PM). Background voxels outside of the brain were not considered in the analysis. The choice of these ROIs was based on the relevance voxel pattern observed in the average heatmaps. Voxel values assigned with 1, 5, 10, and 50% of the positive relevance as well as the whole ROI (100%) were averaged on a subject basis and compared between the contrasts and percentage settings. An example of the ROI setting and the different selected percentages is shown in Figure 2B for a single subject.

**FIGURE 7:**
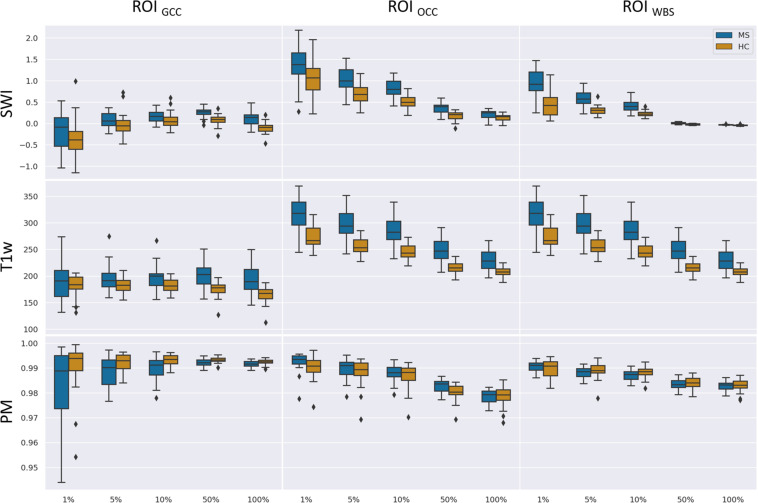
Boxplots of mean values for a given ROI (columns) and number of relevant voxels as a portion of ROI size based on SWI data, T1-weighted data without preprocessing, and PMs.

Figure 7 summarizes the collected data using box plots for all investigated ROI-contrast combinations. For all three ROIs, a difference in SWI and T1w magnitude between the two classes (MS vs. HC) was observed, with values for the MS group being consistently higher on average. This pattern was not observed for the average PM values. Table 3 summarizes this effect in the listed effect sizes (Glass’ Δ), which also takes sample size into account. The results for the PM data (last column in Table 3) showed predominantly negative and smaller values for the effect size in comparison to the SWI and T1w results, and in a number of combinations (7 out of 12), class differences were also found to be not significant (*p* > 0.05, not corrected for multiple comparisons). For the 5 and 10% configurations, the absolute effect size of the PM data was larger for the ROI_*GCC*_ than the absolute effect size of the corresponding T1w data. This may indicate that for this particular ROI (ROI_*GCC*_) PM information contributed more to the relevant voxel in the SWI data, then the magnitude from the T1w data.

**TABLE 3:** Statistical group differences and effect sizes (Glass’ Δ) for the different image contrasts and different ROIs at five percentage configurations (1, 5, 10, 50, 100%).

	SWI		T1w		PM	
			
	*p*-Value	Glass’ Δ	*p*-Value	Glass’ Δ	*p*-Value	Glass’ Δ
1%						
ROI_*GCC*_	0.0725	0.4627	0.2731	0.2323	0.0188	**−0.52**
ROI_*OCC*_	0.0059	0.6809	5.5e-07	**1.2561**	0.0283	0.6568
ROI_*WBS*_	5.0e-08	1.4364	8.7e-10	**1.6031**	0.191	0.4399
5%						
ROI_*GCC*_	0.0338	**0.7076**	0.0337	0.4523	0.0035	−0.6473
ROI_*OCC*_	2.2e-06	1.1975	1.3e-08	**1.4496**	0.2629	0.3382
ROI_*WBS*_	4.1e-10	1.6578	8.5e-11	**1.7416**	0.2454	−0.3673
10%						
ROI_*GCC*_	0.0303	0.6406	0.0072	0.5778	0.0016	**−0.6851**
ROI_*OCC*_	1.4e-08	1.5128	1.9e-09	**1.5656**	0.3429	0.3016
ROI_*WBS*_	1.1e-09	1.5896	7.2e-10	**1.606**	0.0138	−0.6869
50%						
ROI_*GCC*_	4.3e-08	**1.7108**	2.0e-06	1.1537	0.001	−0.7697
ROI_*OCC*_	1.7e-09	**1.7450**	7.9e-09	1.4552	0.001	0.9532
ROI_*WBS*_	7.1e-08	**1.399**	2.2e-05	0.9681	0.3233	−0.2687
100%						
ROI_*GCC*_	2.5e-07	**1.3671**	4.1e-06	1.1025	0.0082	−0.6483
ROI_*OCC*_	0.0059	0.6844	1.4e-06	**1.1340**	0.802	−0.0772
ROI_*WBS*_	0.0002	**1.5166**	0.0003	1.3671	0.5372	−0.1797

**p*-Values for group differences (HC vs. MS) were computed using a two-sample *t*-test. The values with the largest effect size in each ROI-contrast triplet is in bold type.*

## Discussion

In the current study, we introduced a framework for CNN-based MS identification using SWI data. This framework was subsequently examined with regard to the explainability of classification decisions. We applied perturbation analysis to the trained CNN to select an attribution algorithm among three different algorithms based on the quantitative evaluation. Based on the analysis, we used generated DeepLIFT heatmaps to identify important features contributing to the classification task.

In contrast to other MS studies using deep neural networks for disease identification (Eitel et al., 2019), we have chosen SWI because of the evidence of venous patterns in MS in comparison to conventional T2w data. In addition to the venous patterns, SWI can indicate extensive demyelination and iron accumulation. Although deep learning applications can be developed with high performance for MS categorization, which was also shown in this study, the main purpose was to provide new interesting MR based patterns for MS identification, which can then be used as starting points for further analysis. The heatmaps of individual subjects revealed that veins and their surroundings are most relevant for the decision. However, this does not hold for all veins, and there appears to be a preference for certain regions of the brain. These regions are similar for different adjacent slice positions (trained and evaluated completely independent) and are also similar across different echo times. With the current study, we conclude that veins in the anterior medial and lower peripheral regions may be helpful in identifying MS.

**FIGURE 8:**
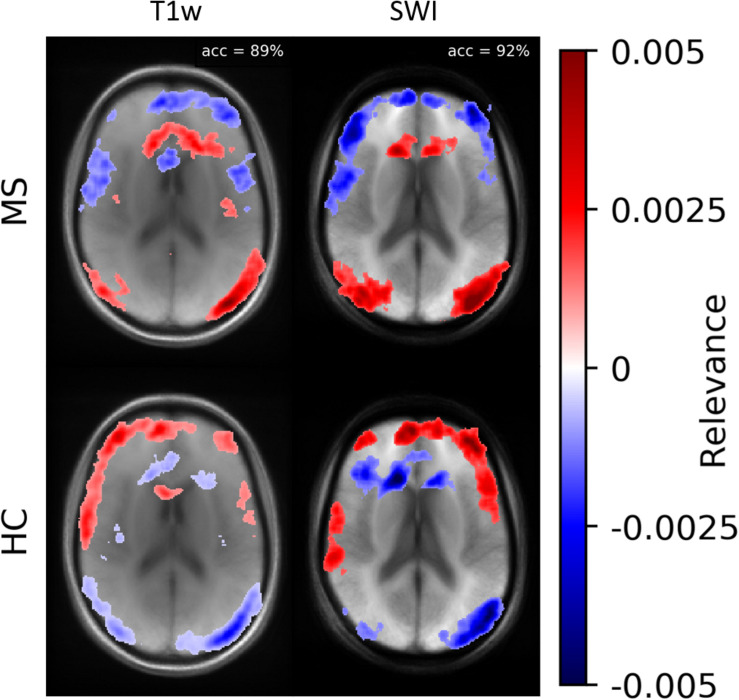
Averaged relevance heatmaps separated for MS (upper row) and HC (lower row) for a network trained and evaluated with T1w (left column) and SWI data (right column).

According to Samek et al. (2017), heatmap information can not only be used for explaining CNNs but also to prioritize image regions and use them for detailed inspection. Thus, we concentrated on ROIs with high heatmap values and used a classical intensity-based analysis. We found numerical differences between the two classes (MS vs. HC) in SWI and T1w data. To check the relevance dependency of SWI data on T1w data, we trained a model based on the T1w data. Figure 8 demonstrates averaged heatmaps for the T1w and the SWI case. The relevance pattern in the anterior medial region differs in detail between contrasts; however, in general, the heatmaps look rather similar. The SWI-based model was found to perform with higher accuracy, which suggests that the PM information added additional identification supporting features to the data.

The classification decision and the corresponding relevance pattern could possibly depend on the demographical attributes of the two groups (MS and HC). To assess the statistical relevance of the findings, we computed *p*-values using a *t*-test for these attributes. The age and gender differences were found to be non-significant (*p* > 0.05).

One limitation of this study is the limited number of samples in the dataset. This circumstance is partly mitigated by using a shallow network and extensive data augmentation during the training procedure. The number of samples (*n* = 132) used in this study is similar to other deep learning studies in detecting neurological disorders (Noor et al., 2020). However, in this study, the pattern relevant for MS identification might only be specific for this limited group of patients. For future studies, we suggest using a larger number of subjects to confirm our results. We also checked the stability of our generated heatmaps by swapping individual datasets between the training and test group and obtained similar heatmap patterns. In addition, the quality of a heatmap depends on the network performance, i.e., a better-trained model provides a useful heatmap that is sparse and less noisy. A second limitation of the study is that the T1w image data used for SWI computation were not corrected for intensity inhomogeneities, which could lead to different results. In combination with the observed averaged intensity differences of relevant voxels between the two classes (MS vs. HC), the question arises whether a class-specific inhomogeneous intensity profile may drive the classification. A brief analysis using intensity corrected magnitude data for SWI computation, network training and evaluation revealed similar locations of relevant voxels (close to veins) for the classification task, but with an overall lower classification performance (∼0.8). Thus, the discrepancy in performance should be investigated in future studies.

Moreover, the choice of the reference input for the DeepLIFT algorithm has an impact on the results. Choosing a useful reference is more intuitive or relies on domain-specific knowledge. Following the recommendation in Shrikumar et al. (2017), we computed DeepLIFT maps against different reference inputs. In our case, we obtained the most promising results with a blurred version of the original input image as a reference. DeepLIFT assigns relevance to the input values, which explains the difference in the output with respect to the reference input values.

A similar attribution analysis of a deep neural network for MS identification has been recently performed by Eitel et al. (2019). This study used a 3D CNN to classify MS patients and healthy controls based on FLAIR contrast and transfer learning. The network was pre-trained on the ADNI MRI data set and fine-tuned on the MS data set. The LRP heatmaps showed that the CNN model was concentrated on posterior periventricular lesions. Compared to Eitel et al. (2019), we focused on 2D SWI data and the DeepLIFT attribution method in the present study. The pattern of most relevant inputs in their LRP-based study differs from data found in our study, which we attribute mainly to the different image contrast.

For future studies, we suggest employing methods of image-to-image-translation (Isola et al., 2017) in order to analyze network decisions in a more human interpretable fashion. With this type of analysis, a successfully trained network for MS identification could be used to perform image modifications to SWI data of a healthy control until the dataset is classified as an MS subject or vice versa. Such an approach might be implemented with StarGAN (Choi et al., 2018) or more specifically using Fixed-Point-GAN (Siddiquee et al., 2019) with image-level annotation during training. However, these new approaches have been only applied for human visible patterns like brain tumors or pulmonary embolism. Thus, it remains currently unclear whether such techniques can be used to identify class-specific patterns in neurological diseases. However, in combination with the results presented in this study the analysis using Fixed-Point GAN might be guided by the obtained averaged heatmaps.

## Conclusion

In the current work, we demonstrated identification of MS patients using a CNN based on 2D SWI data. The subsequent relevance analysis revealed specific areas that were highly relevant for the identification process of the proposed network (the anterior part around the CC and the occipital part). In a simple downstream intensity analysis, we observed statistically significant intensity differences between the two classes in the SWI data. This observation, and the fact that the relevant voxels were mainly located in and around venous vessels, strengthens the presumed association of changes in the vascular system and the development of MS.

## Data Availability Statement

The raw data presented in this article are not readily available due to clinical privacy restrictions. The susceptibility-weighted images and corresponding diagnosis information are available upon request.

## Ethics Statement

The studies involving human participants were reviewed and approved by the Ethics Committee of the Jena University Hospital. The patients/participants provided their written informed consent to participate in this study.

## Author Contributions

AL designed and performed the experiments and wrote the manuscript. SR, RS, and DG organized the database. SR and JR supervised the project. DG designed and supervised the experiments, aided in interpreting results, and writing the manuscript. All authors discussed the results and commented on the manuscript.

## Conflict of Interest

The authors declare that the research was conducted in the absence of any commercial or financial relationships that could be construed as a potential conflict of interest.
